# The role of miR-17-92 in the miRegulatory landscape of Ewing sarcoma

**DOI:** 10.18632/oncotarget.14091

**Published:** 2016-12-22

**Authors:** Raphaela Schwentner, David Herrero-Martin, Maximilian O Kauer, Cornelia N Mutz, Anna M Katschnig, Grzegorz Sienski, Javier Alonso, Dave NT Aryee, Heinrich Kovar

**Affiliations:** ^1^ Children's Cancer Research Institute, St. Anna Kinderkrebsforschung, Vienna 1090, Austria; ^2^ Institute of Molecular Biotechnology of the Austrian Academy of Sciences, Vienna Biocenter Campus, 1030 Vienna, Austria; ^3^ Unidad de Tumores Sólidos Infantiles, Instituto de Investigación de Enfermedades Raras, ISCIII, Ctra, Majadahonda-Pozuelo Km 2, 28220 Madrid, Spain; ^4^ Department of Pediatrics, Medical University, Vienna 1090, Austria; ^5^ Present address: Sarcoma research group, Molecular Oncology Lab, Bellvitge Biomedical Research Institute (IDIBELL), L’Hospitalet de Llobregat 08908, Barcelona, Spain; ^6^ Present address: Whitehead Institute for Biomedical Research, Cambridge, MA 02142

**Keywords:** Ewing sarcoma, EWS-FLI1, miR-17-92, PAR-CLIP, TGFB/BMP pathway

## Abstract

MicroRNAs serve to fine-tune gene expression and play an important regulatory role in tissue specific gene networks. The identification and validation of miRNA target genes in a tissue still poses a significant problem since the presence of a seed sequence in the 3′UTR of an mRNA and its expression modulation upon ectopic expression of the miRNA do not reliably predict regulation under physiological conditions. The chimeric oncoprotein EWS-FLI1 is the driving pathogenic force in Ewing sarcoma. MiR-17-92, one of the most potent oncogenic miRNAs, was recently reported to be among the top EWS-FLI1 activated miRNAs. Using a combination of AGO2 pull-down experiments by PAR-CLIP (Photoactivatable-Ribonucleoside-Enhanced Crosslinking and Immunoprecipitation) and of RNAseq upon miRNA depletion by ectopic sponge expression, we aimed to identify the targetome of miR-17-92 in Ewing sarcoma. Intersecting both datasets we found an enrichment of PAR-CLIP hits for members of the miR-17-92 cluster in the 3′UTRs of genes up-regulated in response to mir-17-92 specific sponge expression. Strikingly, approximately a quarter of these genes annotate to the TGFB/BMP pathway, the majority mapping downstream of SMAD signaling. Testing for SMAD phosphorylation, we identify quiet but activatable TGFB signaling and cell autonomous activity of the BMP pathway resulting in the activation of the stemness regulatory transcriptional repressors ID1 and ID3. Taken together, our findings shed light on the complex miRegulatory landscape of Ewing Sarcoma pointing miR-17-92 as a key node connected to TGFB/BMP pathway.

## INTRODUCTION

Ewing Sarcoma (EwS) is a highly aggressive paediatric cancer and the second most common bone tumour in children and young adults with a peak incidence at the age of 15 [1]. It is characterized by a chromosomal translocation leading to the fusion of EWS and an ETS transcription factor. In 85% of cases the t(11;22)(q24;q12) translocation combines *EWSR1* (Ewing Sarcoma breakpoint region 1) on chromosome 22 with *FLI1* (Friend leukaemia virus integration site 1) on chromosome 11 [2, 3]. This results in a very potent oncogenic transcription factor, EWS-FLI1, comprising the FLI1 ETS DNA binding domain and the transactivation domain of EWS [4].

MicroRNAs (miRs) are short (21–24 nucleotides), single stranded, non-coding RNAs that fine tune gene expression and thus play an important regulatory role in tissue specific gene networks [5]. By binding of the miRNA to a partially homologous sequence (seed region) mostly located within the 3’ untranslated region (UTR) of a transcript, it can either block its target mRNA translation or lead to its degradation [6, 7]. As a consequence of imperfect base pairing of the miRNA to its seed region, a single miRNA can regulate many different target mRNAs resulting in a complex network of miRNAs and their targets [5, 8]. It is estimated that up to 60% of the human genome is regulated by miRNAs affecting genes involved in regulation of normal and pathological cellular functions such as proliferation, inflammation, stress response, apoptosis, differentiation and invasion [5, 7].

The complexity of miRNA-mRNA interactions is one of the main reasons why the identification and validation of miRNA target genes in a given tissue still poses a significant problem [9]. Furthermore, algorithms predicting miRNA-mRNA interactions solely based on sequence matching are insufficient and additional parameters such as orthologous sequence alignment, UTR context, free energy of complexes or sequence conservation have to be considered [9]. However, despite the progress made during the last years, current prediction tools still have suboptimal performance with both high false-positive and false-negative rates [10]. In addition, the activity of an expressed miRNA on a putative target mRNA depends on their relative abundances in comparison to other co-expressed putative target mRNAs in a tissue specific manner, and several expressed pseudogenes and non-coding RNAs may act as competing endogenous RNA (ceRNA) for miRNA function [11]. Therefore, approaches applying forced expression of ectopic miRNA mimics to define targets for miRNAs of interest are of limited relevance, since they may only identify potential targets but do not allow prediction of their miRNA dependent regulation under physiological conditions. Still, this approach is most frequently used for miRNA target validation.

In our efforts to define the role of specific miRNAs in Ewing sarcoma, we therefore sought to apply a strategy which directly identifies the miRNA-mRNA complexes in the tissue context by identifying target transcripts associated with the functional RNA-induced silencing complex (RISC) [10]. Hafner et al. first described PAR-CLIP (Photoactivatable-Ribonucleoside-Enhanced Crosslinking and Immunoprecipitation) in 2010 as a method relying on the incorporation of photoreactive ribonucleoside analogs (i.e. 4-thiouridine) into transcribed RNA, which are subsequently cross-linked to associated RNA binding proteins by UV. After immunoprecipitation with antibodies to the protein of interest (i.e. the miRNA-binding RISC component AGO2), the cross-linked RNA is isolated and sequenced. The exact sites of miRNA-mRNA interaction at the sites of AGO2 crosslinks are revealed by thymidine to cytidine transitions in the cDNAs prepared from immunopurified RISC complexes of 4-thiouridine-treated cells [12]. Using this approach, we defined the full complement of RISC associated miRNA-mRNA complexes in the EwS cell line A673/TR/shEF. Subsequently, we used miRNA sponges to probe regulation of mRNA targets identified by PAR-CLIP for miRNAs of interest. MiRNA sponges comprise multiple tandem arrayed seed sequences for specific miRNAs that compete for miRNA binding when ectopically expressed from a strong promoter. MiRNA with a complementary heptameric seed are specifically inhibited; thereby a single sponge can be used to block an entire miRNA seed family [13] (for review see [14, 15]).

Recently, we reported on the EWS-FLI1 miRNA signature based on knockdown experiments in EwS cell lines and on differential expression in primary tumours versus mesenchymal stem cells (MSC). Comparison of both datasets revealed the hsa-miR-20a-3p to be the top EWS-FLI1 activated miRNA [16]. In addition, conditional knockdown of EWS-FLI1 in the EwS cell line A673/TR/shEF revealed co-regulation of the entire set of miRNAs of the hsa-miR-17-92 cluster. This miRNA cluster maps to human chromosome 13 and encodes for six individual miRNAs (hsa-mir-17, hsa-miR-18a, hsa-miR-19a, hsa-miR-19b-1, hsa-miR-20a and hsa-miR-92-1), which group into four “seed” families (hsa-miR-17/20a, hsa-miR-18, hsa-miR-19ab and hsa-miR-92) [17–19]. The hsa-miR-17-92 cluster, also called OncomiR-1, encodes the most potent oncogenic miRNAs and was first described to be amplified in B cell lymphoma [20, 21]. Until now, overexpression of OncomiR-1 was reported in a variety of solid and haematological cancers including T-cell leukaemia, neuroblastoma, and medulloblastoma [19]. The whole cluster is among the small number of miRNAs down-regulated upon knockdown of EWS-FLI1 in multiple EwS cell lines [16].

In this study we used a combination of AGO2 pull-down experiments by PAR-CLIP and miRNA depletion by ectopic sponge expression followed by RNA-sequencing (RNAseq), to identify the full targetome of hsa-miR-17-92 in EwS cells. We found a significant enrichment of PAR-CLIP hits for members of the miR-17-92 cluster in the 3′UTRs of genes up-regulated in response to hsa-mir-17-92 specific sponge expression. Among them, we consistently identified known and a multitude of so far unknown targets of the OncomiR-1 cluster in EwS. Strikingly, approximately a quarter of these genes annotate to the TGFB/BMP pathway, the majority mapping downstream of SMAD signaling.

## RESULTS

### hsa-miR-17-92 is among top EWS-FLI1 activated miRNAs

**Figure 1:**
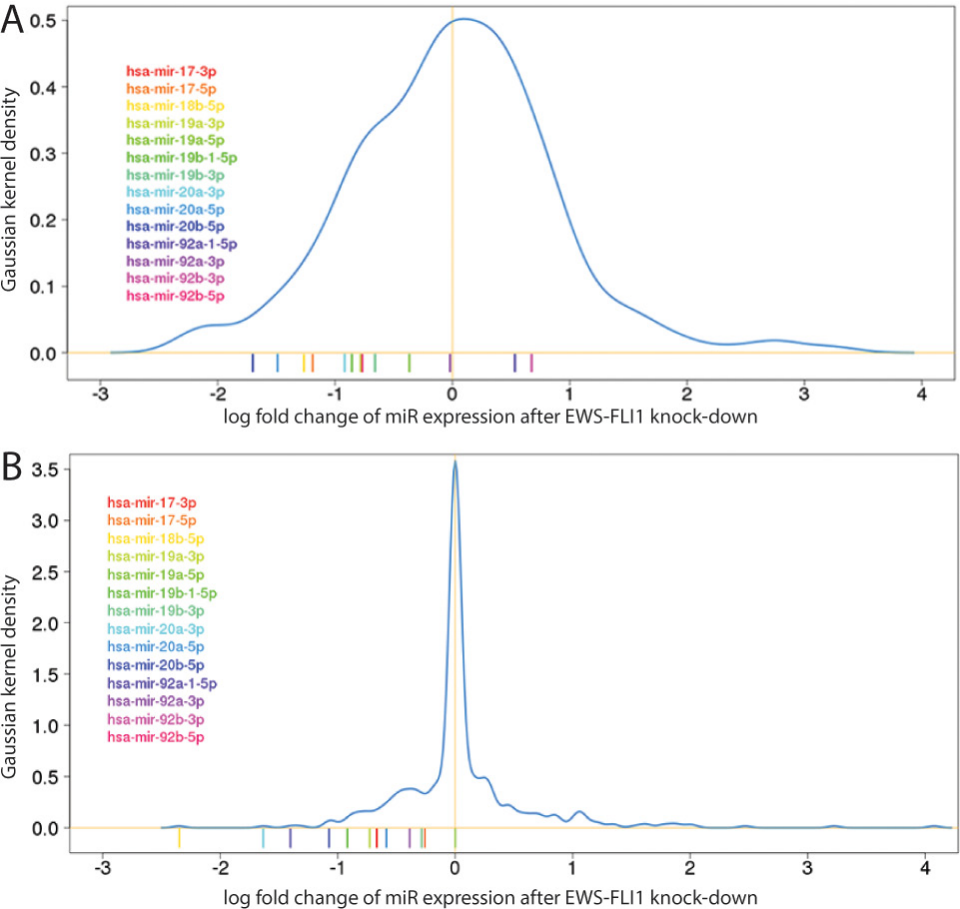
Histograms of differential miR expression levels (x-axis: log-2 fold change, y-axis: Gaussian kernel density) after EWS-FLI1 knockdown from (**A**) miRNA-Seq in the A673/TR/shEF cell line after doxycycline inducible knockdown of EWS-FLI1 (**B**) mean from transient knockdown experiments of EWS-FLI1 in 5 different EwS cell lines measured on qPCR based platform [16]. The coloured bars below the histogram indicate miRNAs from the 17-92 cluster.

Previously, we assessed miRNA expression on a qPCR platform in five EwS cell lines following sh-RNA mediated knockdown of EWS-FLI1 [16]. This data revealed that members of the hsa-miR-17-92 cluster were among the most highly down-regulated miRNAs after the EWS-FLI1 knockdown. Taking an average across cell lines, particularly hsa-miR-17, hsa-miR-18a/b, hsa-miR-19a/b and hsa-miR-20a/b were among the top 30 most down-regulated genes in these experiments. This finding was corroborated in A673/TR/shEF EwS cells by miR-seq after doxycycline inducible knockdown of EWS-FLI1 (Figure 1, Supplementary Table S1). Thus the hsa-miR-17-92 cluster is activated by EWS-FLI1 and is therefore of prime interest for studying miRNA mediated effects of EWS-FLI1 in EwS.

### Genes identified by PAR-CLIP are hsa-miR-17-92 targets

**Figure 2:**
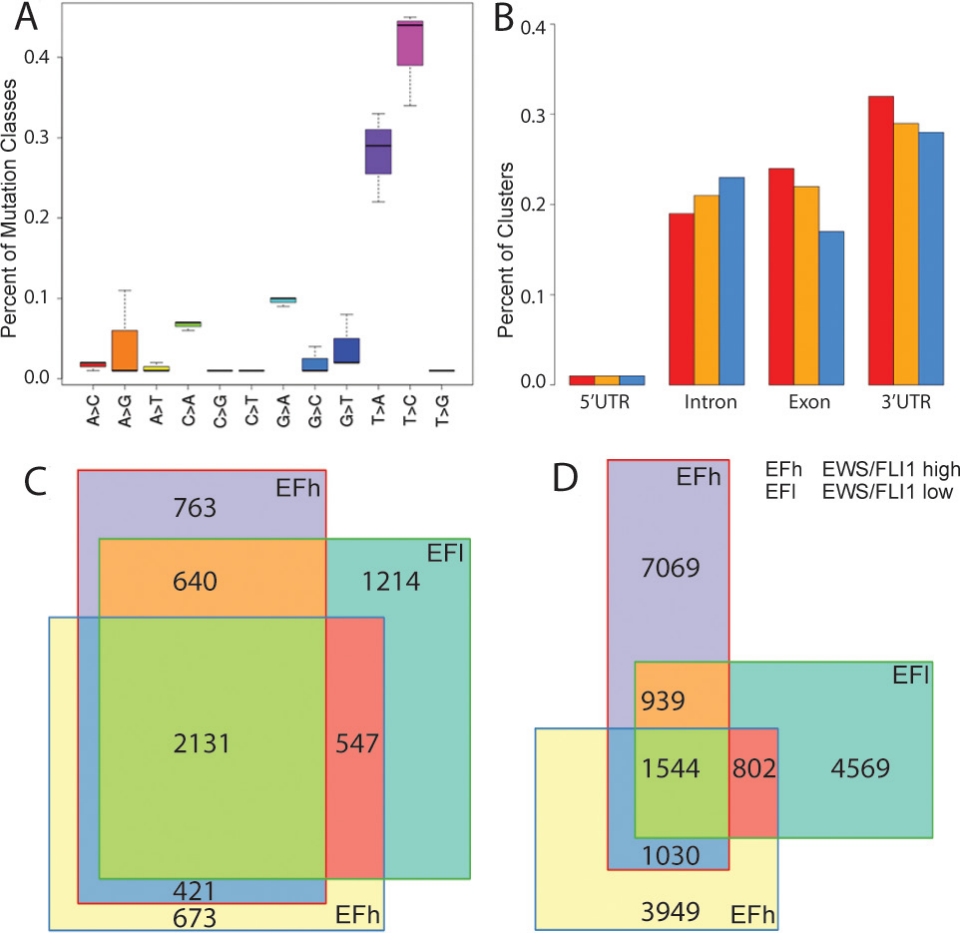
PAR-CLIP results (**A**) Percentage (y-axis) of the mutation classes found in 3 replicates (boxes) of the PAR-CLIP experiment. (**B**) Percent AGO2 occupancy of RNA in different regions of genes for clusters in the PAR-CLIP data called by PARalyzer [22]. The 3 replicas are shown in different colours. Number of overlapping genes (**C**) and overlapping PARalyzer clusters (**D**) for the three AGO2 PAR-CLIP replica experiments.

To characterize the full complement of endogenous miRNA-mRNA complexes in A673/TR/shEF EwS cells, we performed PAR-CLIP under EWS-FLI1 high and low conditions monitoring target transcripts associated with the RNA-binding component of the functional RISC complex, AGO2. Indicative of specific AGO2/mRNA cross linking, a high T to C conversion rate of at least 35% was observed in all samples (as compared to 10–20% previously reported by Hafner et al. for non-irradiated 4SU-containing oligoribonucleotides (Figure 2A) [12]. Also, 3’UTRs were highly overrepresented in the clusters identified by PARalyzer, a versatile tool for mapping high-confidence sites from PAR-CLIP deep-sequencing data [22] (Figure 2B). Overall, 7860-10601 clusters from PARalyzer mapped to 3′UTRs and also fulfilled further quality filters (Supplementary Table S2, see M&M). These clusters were contained in 3955-4532 genes. 2131 of these genes had PARalyzer clusters in all three experiments, and 1583 clusters overlapped exactly (min 10bp overlap). Interestingly, concerning pairwise overlaps, the two replicas without knockdown of EWS-FLI1 had a very similar percentage of shared genes (64%) and clusters (32%) as each of these experiments with the EWS-FLI1 knockdown experiment (genes: 53%/70%, clusters:32%/35%, calculated with respect to the smaller set, Figure 2C, 2D). This result indicated that the silencing of EWS-FLI1 did not have a large influence on the spectrum of AGO2 binding sites on mRNAs as identified by PAR-CLIP. Since the method does not allow drawing any quantitative conclusions, we did not distinguish between the two conditions and treated them as replicates for the remainder of this study

### MiRNA seeds in PAR-CLIP clusters

The 2131 genes that harboured PAR-CLIP clusters in all three replicas were analysed for overrepresentation of miR seeds in 3′UTRs of genes as annotated in the MSigDb (http://software.broadinstitute.org/gsea/msigdb). The hsa-miR-17-92 cluster was found to be most significantly overrepresented in this gene set (Supplementary Table S3). Similarly, among the top 10 miRNA seeds found in the clusters that overlap in all three PAR-CLIP experiments were seeds matching the hsa-miR-17-92 cluster and hsa-miR-19a/b-3p: AAAGTGC(7mer), TAAAGTGC(7mer1A), GTGCAAA(7mer).

### Integration of PAR-CLIP and hsa-miR-17-92 sponge RNA-Seq

The hsa-miR-17-92 miRNA cluster encodes for six individual miRNAs. To deplete the entire miRNA family we used a miRNA sponge specific to hsa-miR-17-92 (s-α-hsa-miR-17-92) followed by RNA-seq to determine mRNAs regulated by hsa-miR-17-92. As negative controls, a non-targeting miRNA sponge based on a sequence from CXCR4 devoid of any miRNA seed motifs and a sponge against hsa-miR-9 (s-α-hsa-miR-9) were used, as this miRNA is moderately expressed in A673/TR/shEF (7-fold less than the highest expressed hsa-mir-17-92 family member hsa-mir-20a-5p ).

Differential expression analysis identified 256 genes specifically down-regulated and 182 genes up-regulated after treatment with s-α-hsa-miR-17-92(|fold-change| > 1.5, FDR < 0.1). There was no significant overlap with genes up- or down-regulated by s-α-hsa-miR-9 (Supplementary Table S4, Sheet1).

Intersection of RNA-seq and PAR-CLIP data was performed, so that for every gene the log fold expression change after sponge treatment and the presence or absence of PAR-CLIP hits and seed sequences therein in the 3′UTR of that gene was recorded. With this data set, all annotated seed sequences were tested for overrepresentation in the genes up-regulated after sponge treatment. Furthermore, PAR-CLIP hits were only counted if they were found in at least two of the replicas. As miRNAs most often have a relatively mild effect on the abundance of mRNAs [23], we chose a non-stringent cutoff for differential gene expression in this analysis (log2fold-change > 0.3). In this analysis, seeds closely matching the hsa-miR-17-92 seed sequence (8mer: AAGTGCAT, 7mer: AAAGTGC, 7mer1A: AAGTGCA) were found to be significantly overrepresented (*P* < 0.001, hypergeometric test) in genes up-regulated after s-α-hsa-miR-17-92 application.

The intersection of PAR-CLIP and s-α-hsa-miR-17-92 overexpression data yielded a list of 87 genes that were up-regulated upon s-α-hsa--miR-17-92 treatment and also had a PAR-CLIP hit with a seed sequence matching hsa-miR-17-92 (AAGTGCAT: 8mer for hsa-miR-18a-5p, hsa-miR-18b-5p, AAAGTGC: 7mer for miR-17-5p, hsa-miR-20a-5p, hsa-miR-106a-5p, hsa-miR-106b-5p, hsa-miR-20b-5p) (Supplementary Table S4, Sheet2).

Pathway analysis of this gene list using DAVID (https://david.ncifcrf.gov) [24] and the MSigDB (http://software.broadinstitute.org/gsea/msigdb) [25], identified members of several transcriptional pathways, including the insulin receptor, p53, Wnt, TGFB, respectively SMAD2/3 pathways (Supplementary Table S4, Sheet 3, 4). Although only a few genes were annotated to these pathways, five pathways in the MSigDB showed significant enrichment (Table 1). However, manual curation of the list of 87 genes revealed that 20 of these were found in the literature to be linked (mostly downstream) to the TGFB/BMP pathway confirming a previously proposed link between hsa-miR-17-92 and TGFB signaling observed in neuroblastoma [26, 27] (see Supplementary Table S5).

**Table 1:** Pathways significantly enriched

Gene Set Name	# Genes in Gene Set (K)	Description	# Genes in Overlap (k)	k/K	*p*-value	FDR *q*-value
REACTOME_GENERIC_TRANSCRIPTION_PATHWAY	352	Genes involved in Generic Transcription Pathway	10	0.0284	1.45E-09	1.93E-06
PID_SMAD2_3NUCLEAR_PATHWAY	82	Regulation of nuclear SMAD2/3 signaling	4	0.0488	1.87E-05	1.24E-02
REACTOME_PPARA_ACTIVATES_GENE_EXPRESSION	104	Genes involved in PPARA Activates Gene Expression	4	0.0385	4.77E-05	1.75E-02
REACTOME_TRANSCRIPTIONAL_ACTIVITY_OF_SMAD2_SMAD3_SMAD4_HETEROTRIMER	38	Genes involved in Transcriptional activity of SMAD2/SMAD3:SMAD4 heterotrimer	3	0.0789	5.27E-05	1.75E-02
PID_TGFBR_PATHWAY	55	TGF-beta receptor signaling	3	0.0545	1.60E-04	4.26E-02

### Target gene validation

We found a significant enrichment of PAR-CLIP hits for members of the hsa-miR-17-92 cluster in the 3′UTRs of genes up-regulated in response to hsa-mir-17-92 specific sponge expression. Among them we identified known (i.e. *CTGF, MXD1, BAMBI, ABL2, KDM2A, CDKN1A, MAPK8*, Supplementary Table S6) and a multitude of so far unknown targets for this cluster in three PAR-CLIP experiments [28–34].

**Figure 3:**
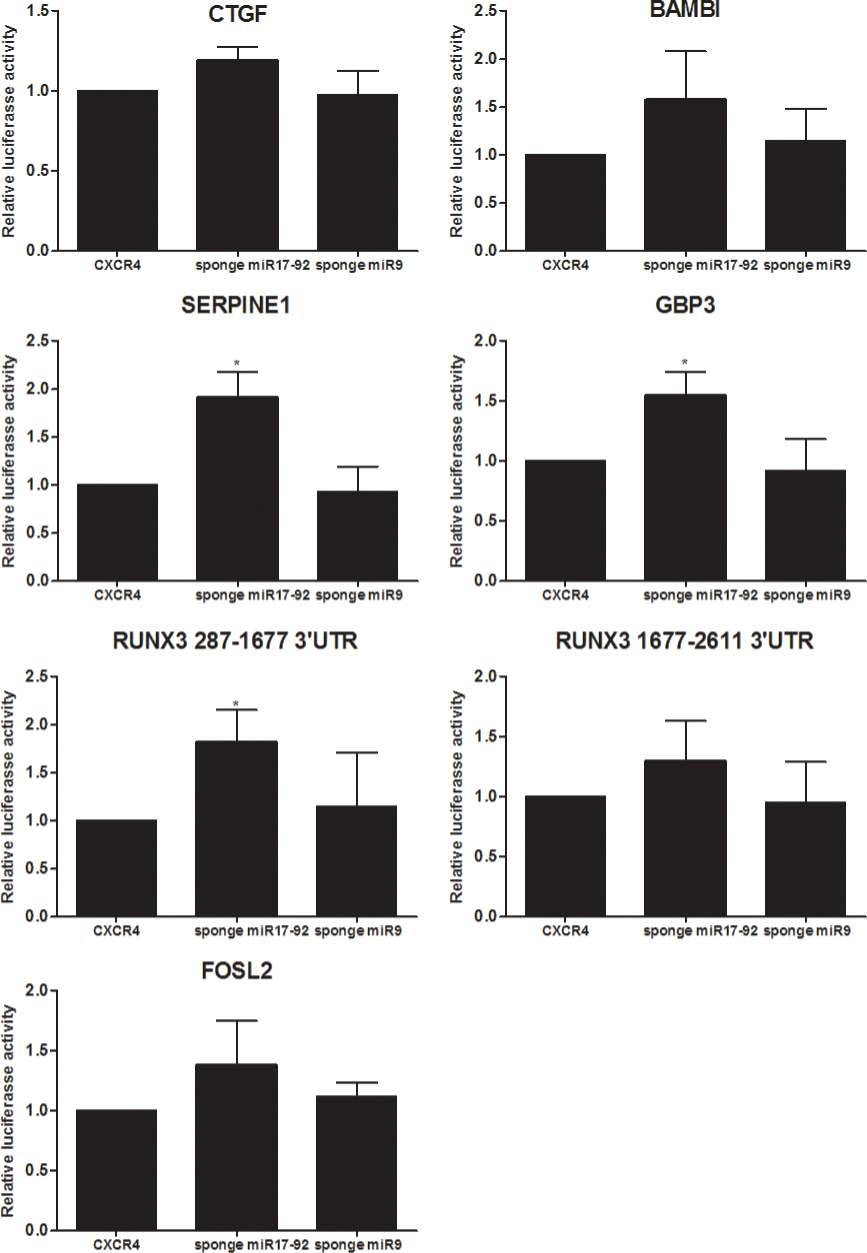
Target gene validation by luciferase assays Firefly luciferase reporter assays for 6 genes which harbour a PAR-CLIP hit and which were found to be up-regulated upon introduction of a hsa-miR-17-92 specific sponge. 3′UTR fragments of CTGF, FOSL2, SERPINE1, BAMBI, GBP3 and RUNX3 (two parts: 287-1677 and 1677–2611) were cloned into the pmirGLO vector (Promega) and tested for responsiveness to expression of either a sponge to hsa-miR-17-92 or low expressed hsa-miR-9, as well as a control 3′UTR of CXCR4 devoid of any miRNA seed sequences. The y-axis represents the promoter activity relative to control conditions (CXCR4). Means and standard deviations of at least three independent experiments, each performed in triplicate, are shown.

Of those genes identified by PAR-CLIP to be targeted by a miRNA and up-regulated in response to sponges for hsa-miR-17-92, but not for hsa-miR-9, the 3′UTRs of six genes were further functionally studied as miRNA targets in luciferase reporter assays. The full length 3′UTR of *CTGF, FOSL2, BAMBI, SERPINE1, GBP3* and two fragments of *RUNX3* were co-transfected with either the 3′UTR of *CXCR4*, or sponges against hsa-miR-17-92, or, as control, hsa-miR-9 (Figure 3). While the well described hsa-miR-17-92 targets *CTGF* and *BAMBI* showed only moderate induction upon overexpression of the corresponding sponge, luciferase activity was significantly increased for *SERPINE1* and *GBP3* 3′UTR constructs [30, 35].

The 3′UTR of *RUNX3* was cloned in two parts, one spanning basepairs 287-1677 of the 3′UTR containing a hsa-miR 17/20a seed sequence identified by PAR-CLIP, and a second, spanning bp 1677 to 2611 containing a hsa-miR-17 and -19ab seed sequence identified by TargetScan. Contrary to the significant increase of *RUNX3* 287-1677 luciferase activity upon administration of s-α- hsa-miR-17-92, *RUNX3* 1677-2611 luciferase activity was only moderately increased. A similar increase was observed for *FOSL2*. Unlike the significant or at least moderate induction of luciferase activity of all above mentioned 3′UTR constructs as a consequence of s-α-hsa-miR-17-92 expression, forced expression of s-α-hsa-miR-9 had no effect. This is in accordance with our previous RNA-seq results and further emphasizes the fact that hsa-miR-17-92 decreases the activity of those genes, while hsa-miR-9 does not.

**Figure 4:**
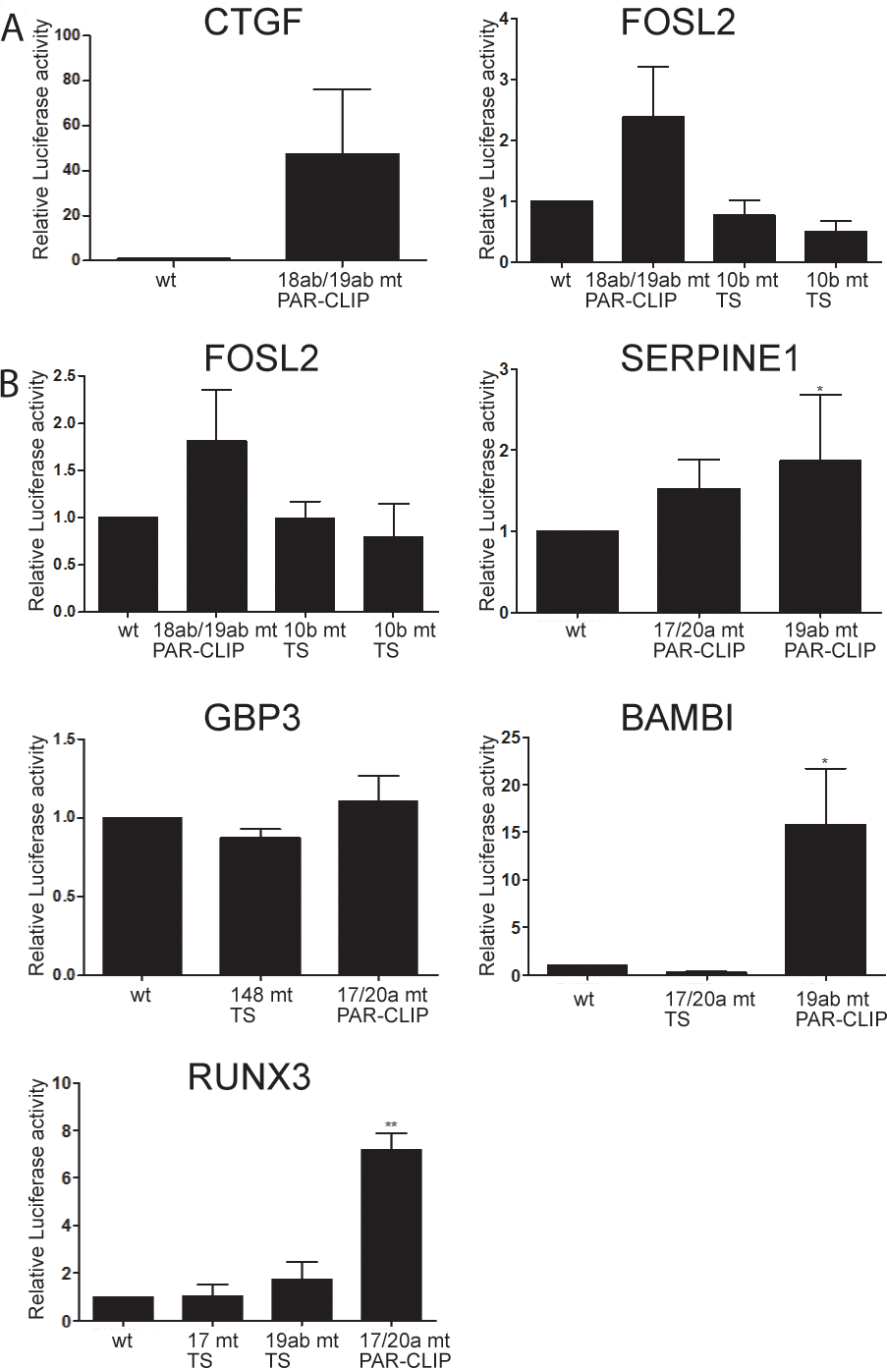
Mutation of hsa-miR-17-92 seed sequences in putative target 3′UTRs results in up-regulation of reporter activity (**A**) Luciferase expression constructs with full length 3′UTRs of CTGF and FOSL2 were transfected either in wildtype configuration or with mutations in miRNA seed sequences (CTGF 18ab/19ab, FOSL2 18ab/19ab and two individual 10b seed sequences), and were analyzed by reporter assays. (**B**) Oligonucleotides of about 40bp length harbouring miRNA seed sequences identified by PAR-CLIP or by TargetScan were cloned 3′ to luciferase in the pmirGLO plasmid, and reporter activity was compared between constructs with wildtype and mutated miRNA seed sequences. The y-axis represents the promoter activity relative to control conditions (wt seed sequences). PAR-CLIP: seed sequences identified by PAR-CLIP, TS: seed sequences predicted by TargetScan. Means and standard deviations of at least three independent experiments, each performed in triplicate, are shown.

To further validate the specific effect of hsa-miR-17-92 on the 3′UTRs of these known and novel targets, we mutated the miRNA seed sequences in *CTGF* and *FOSL2* (Figure 4A). Mutation of the hsa-miR-18ab/19ab seed sequences (identified by PAR-CLIP) in the *CTGF* 3′UTR caused on average a 40 fold induction of luciferase activity. For the study of the *FOSL2* 3′UTR, mutations were introduced at several locations. First, the hsa-miR-18ab/19ab seed sequence identified by PAR-CLIP was abrogated leading to an increase in luciferase activity of about 2 fold. Second, two independent hsa-miR-10b (which is expressed at similar levels as miR-17-92) seed sequences, solely identified by TargetScan, were altered without any effect. Similar results were obtained with wildtype and miRNA seed sequence mutated 40mer oligonucleotides cloned in pmirGLO (Figure 4B).

The 3′UTR of *SERPINE1* contains two miRNA seed sequences detected by PAR-CLIP. Mutations at both sites raised reporter activity, yet only the increase obtained by destruction of the hsa-miR-19ab seed sequence achieved significance. In the 3′UTR of *GBP3* neither mutation of the hsa-miR-148 seed (predicted by TargetScan), nor the hsa-miR-17/20a seed sequence displaying AGO2 interaction in PAR-CLIP affected luciferase activity. Unlike the minor increase in signal intensity of the 3′UTR fragment of *BAMBI* with s-α- hsa-miR-17-92, destruction of the hsa-miR-19ab recognition site identified by PAR-CLIP caused a significant 15fold increase in luciferase activity. Again mutation of a hsa-miR-17/20a seed sequence predicted by TargetScan but not detected by PAR-CLIP did not alter luciferase signal intensity. In line with these results mutation of a hsa-miR-17/20a PAR-CLIP seed sequence in the 3′UTR of *RUNX3* increased luciferase activity by approximately 7 fold, whereas mutation of a hsa-miR-17/20a and a hsa-miR-19ab exclusively TargetScan defined seed sequence did not significantly increase luciferase activity. These results confirm the superiority of our combined PAR-CLIP/sponge expression/RNAseq approach over in-silico prediction to identify directly miRNA regulated targets in a tissue specific context, and identifies a large number of novel OncomiR-1 regulated genes in EwS.

**Figure 5:**
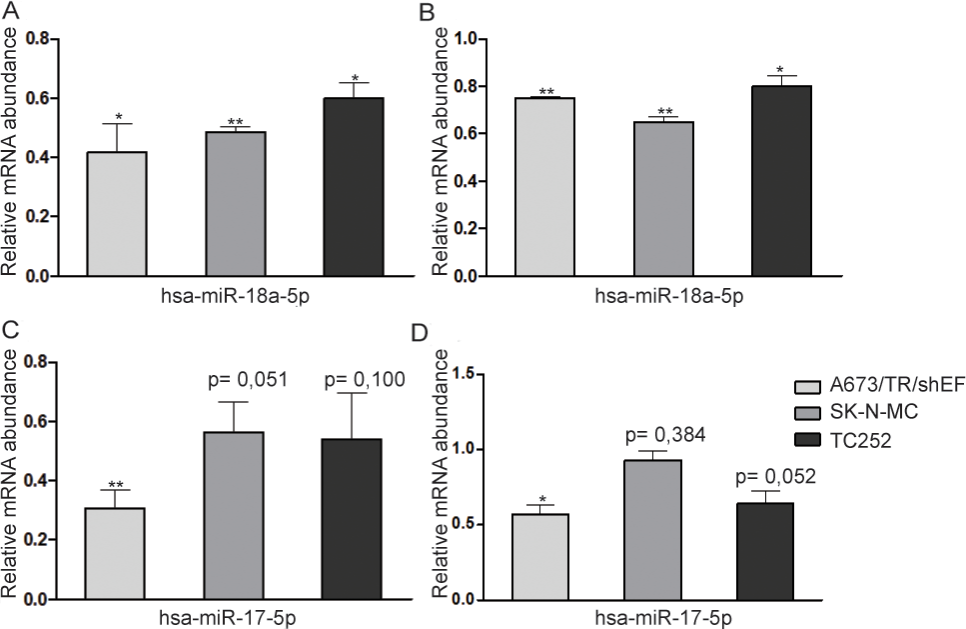
miRNA overexpression in three EwS cell lines miRNA mimics (hsa-miR-18a-5p and hsa-miR-17-5p) and a negative control mimic were transfected in A673/TR/sheEF, SK-N-MC and TC252 EwS cell lines, and qPCR was performed for (**A**) CTGF, (**B**) FOSL2, (**C**) GBP3 and (**D**) SERPINE1. Relative mRNA abundance is calculated against negative control mimic. Means and standard deviations of at least three independent experiments, each performed in triplicate, are shown.

Finally, we validated our data for four arbitrarily chosen targets (*CTGF*, *FOSL2*, *GBP3*, *SERPINE1*) in A673/TR/shEF and two additional EwS cell lines, SK-N-MC and TC252 using conventional ectopic miRNA mimic expression, followed by qPCR (Figure 5). In agreement with our results from PAR-CLIP and gene reporter assays, mRNA levels of all four tested genes were significantly reduced upon transfection of miRNA-mimics (hsa-miR-18a-5p for *CTGF* and *FOSL2* and hsa-miR-17-5p for *GBP3* and *SERPINE1*) in A673/TR/shEF compared to a negative control mimic transfected in parallel. Overexpression of miRNA mimics in SK-N-MC caused a highly significant reduction of mRNA levels for *CTGF* and *FOSL2*, and a marginally significant reduction of 40% for GBP3. A similar result was obtained in TC252, *CTGF* and *FOSL2* mRNA expression was significantly reduced upon transfection of miRNA mimic hsa-miR-18a-5p compared to the negative control, while *SERPINE1* was marginally statistically significant (*p* = 0,052). Overall these results further confirmed our previously generated data and expanded it to two additional cell lines.

### Activity of TGFB and BMP pathways in Ewing sarcoma cell lines

**Figure 6:**
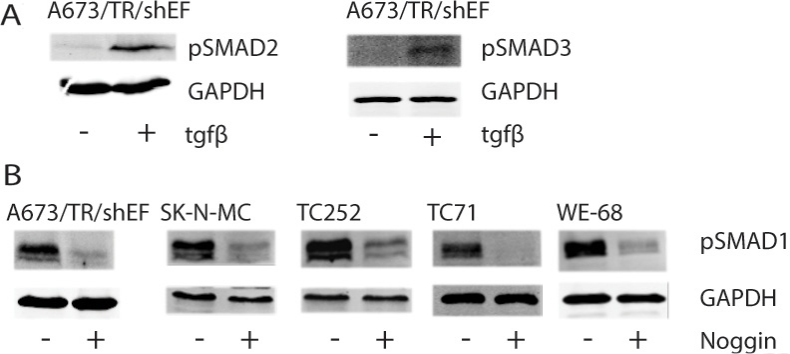
Phosphorylation status of SMADs upon TGFB or Noggin treatment Cells were either left untreated or treated (+) with (**A**) 5 ng/ml TGFB for 1h or (**B**) 300 ng/ml Noggin overnight. Protein levels of (A) GAPDH, pSMAD2, pSMAD3 and (B) pSMAD1 were monitored by fluorescent immunoblotting using the LICOR Odyssey® Infrared Imaging System.

Since we found a marked enrichment of TGFB/BMP pathway components among hsa-miR-17-92 targets, we tested for SMAD2/3 and SMAD1 phosphorylation as read-out of TGFB and BMP signaling in EwS cell lines, As shown in Figure 6A for A673/TR/shEF cells, no SMAD2 and SMAD3 phosphorylation was observed under standard growth conditions, consistent with the previously reported suppression of TGFB activity by EWS-FLI1 in EwS. [36]. However, upon ectopic ligand administration, a clear SMAD2/3 phosphorylation signal was observed, suggesting that the TGFB pathway is not irreversibly blocked by the oncogene and responsive to micro-environmental signaling cues. In contrast, SMAD1 phosphorylation was consistently observed in all five EwS cell lines tested (A673/TR/shEF, SK-N-MC, TC252, TC71, WE-68) (Figure 6B), even in the absence of serum (not shown). Addition of the polypeptide BMP inhibitor Noggin blocked SMAD1 phosphorylation indicative of constitutively active, cell autonomous BMP signaling in EwS.

To test the functional consequence of active BMP signaling in EwS cell lines, we performed RNAseq under control and Noggin treated conditions in A673/TR/shEF, SK-N-MC, and TC252 cells. Sixteen hours of Noggin treatment resulted in down-regulation of ID1 and ID3 mRNAs as the most immediate significant changes in gene expression in all three cell lines tested (Supplementary Table S7). Consistent with this finding, GSEA at this early time point of BMP suppression by Noggin identified enrichment of a MSC signature in two of the cell lines, suggesting a role for BMP signaling in the regulation of EwS differentiation (Supplementary Figure S1).

## DISCUSSION

The unambiguous, unbiased identification of miRNA-mRNA interactions and corresponding post-transcriptional gene regulation in a specific tissue context under physiological conditions remains difficult. In this study, we addressed this problem by a combination of physical interaction studies between miRNAs and their target mRNAs in the RISC complex as revealed by PAR-CLIP and monitoring of genome wide expression responses to competitive miRNA depletion by forced miRNA sponge expression. Although the strong enrichment of 3′UTRs and of miRNA seed sequences in the immediate vicinity of AGO2 interaction sites suggested high specificity and sensitivity of PAR-CLIP to identify miRNA-mRNA interactions on a genome-wide scale, it was not sufficient to predict miRNA dependent target regulation. Despite a significant overlap between target spectra obtained from three independent PAR-CLIP experiments, there was considerable quantitative variation in read counts unrelated to presence and absence of EWS-FLI1, which we and others had previously demonstrated to shape the EwS miRNome [16, 37–40]. In addition, association with the RISC complex did not allow prediction of modulation of target mRNA levels at all. This may in part be due to the problem that, while RISC association is a prerequisite for miRNA mediated mRNA degradation, efficient degradation is predicted to lead to a depletion of the target sequence in AGO2 precipitates. Also, miRNA mediated gene regulation is considered a rheostat rather than an on/off switch of gene expression, and thus, under physiological conditions, miRNA effects on target mRNA levels may be subtle [41]. Therefore, we combined PAR-CLIP with a functional read-out, the response of gene expression to competitive miRNA depletion by the introduction of specific miRNA sponges. We chose sponges to modulate miRNA availability. We demonstrate for members of the hsa-miR-17-92 cluster that our approach is suited to identify known and many novel miRNA targets. By mutation analysis of miRNA seed sequences in reporter gene assays performed for a series of newly identified hsa-miR-17-92 targets, we functionally validated their regulation via miRNA interaction sites characterized by PAR-CLIP. These experiments also demonstrated for selected genes (i.e. *BAMBI* and *RUNX3*) that target sites defined exclusively by in silico prediction (i.e. TargetScan) but not by PAR-CLIP were functionally inactive in reporter assays. Together, these results identify a combination of PAR-CLIP and RNAseq after miRNA depletion by sponges as a highly efficient approach to identify miRNA regulated genes in their specific cellular environment.

Intersecting PAR-CLIP and RNAseq datasets we obtained a list of putative hsa-miR-17-92 directly regulated target genes. Functional annotation revealed that about a quarter of these genes annotate to the TGFB/BMP pathways, mainly downstream of SMAD signaling. These pathways play crucial roles in embryonic development, adult tissue homeostasis and, if deregulated, in the pathogenesis of a variety of diseases. Upon ligand binding, activated TGFB or BMP receptor complexes phosphorylate the carboxy-terminus of receptor-regulated SMAD proteins, including SMAD1, 5 and 8 for BMP and SMAD2 and 3 for TGFB signaling [42]. In addition to the canonical SMAD-dependent pathways, non-canonical TGFB/BMP signaling acts through activation of the p38 mitogen-activated protein kinase pathway. Both signal transduction mechanisms regulate RUNX2, which is essential for skeleton formation and osteoblast gene expression and differentiation [43].

Previously, the hsa-miR-17-92 cluster was reported to directly target the TGFB/BMP pathway in neuroblastoma. Here, activation of hsa-miR-17-92 caused an increase in *TGF-β* receptor 2 (*TGFBR2), SMAD2* and *SMAD4* expression levels, and mutations of the corresponding seed sequences indicate that hsa-miR-17 and hsa-miR-20a directly target *TGFBR2*, whereas hsa-miR-18a was found to modulate SMAD2 and SMAD4. In addition, not only key effectors along the TGFB pathway were affected, but also downstream targets as *CDKN1A*, *SERPINE1* and *BCL2L11* [26, 27]. This multi-layered regulation of TGFB signaling by targeting several up- and downstream pathway components through multiple miRNAs of the same cluster enables flexible but precise pathway regulation. Our analysis in EwS independently confirms some of the targets identified in neuroblastoma and discovers a number of previously unknown targets related to TGFB/BMP signaling.

In EwS, the TGFB/BMP pathway is considered to be inactive, as *TGFBR2* is strongly and directly repressed on the transcriptional level by EWS-FLI1 [44]. Yet, our RNAseq and gene expression data imply that, at least in A673/TR/shEF cells, *TGFBR2* is not completely off and TGFB1 and TGFB2 ligands are expressed at low levels [45, 46]. Pardali et al. reported that endoglin, a TGFB co-receptor, is present in EwS and correlates with dismal prognosis of EwS patients. Down-regulation of endoglin caused reduced tumour growth in xenograft mouse models. Furthermore, evidence was provided that endoglin attenuated TGFB responsiveness, but was required for BMP signaling [47]. Our observations in A673/TR/shEF cells may corroborate some of these findings in that we found SMAD2 and SMAD3 not phosphorylated under standard growth conditions, but remaining weakly responsive to treatment with TGFB. By contrast, we observed cell autonomous constitutive SMAD1 phosphorylation resulting in activation of ID1 and ID3 transcriptional repressors. Consistent with this finding, GSEA revealed enrichment of the TGFB pathway among Noggin suppressed gene sets in all three cell lines. Among activated gene sets, we found enrichment of a stromal stem cell signature in two of the cell lines (A673/TR/shEF and SK-N-MC) and M-G1 cell cycle transition in one cell line (TC252) (Supplementary Figure S1). This finding is consistent with previous findings from osteosarcoma which demonstrated that *ID1* and *ID3* stabilize a MSC program, suggesting that BMP signaling may regulate stemness in Ewing sarcoma as well [48].

EwS most frequently presents as a poorly differentiated small round cell tumour of the bone that is considered to originate from MSC [49, 50]. TGFB/BMPs promote differentiation of human MSC into the osteogenic lineage via SMAD mediated activation of RUNX2 [43]. One of the hsa-miR-17-92 targets confirmed in our study is BAMBI, a pseudo-receptor of the BMP signaling pathway related to TGFB family type I receptors lacking an intracellular kinase domain. It associates with TGFB family receptors and ligands and thereby inhibits BMP signaling [51]. Recently it was shown that hsa-miR-20a directly targets BAMBI in human MSC promoting osteogenic differentiation [30]. Our data confirm BAMBI as a target of hsa-miR-17-92 in EwS, consistent with the observed constitutive BMP signaling. However, data from the mouse myoblast cell line C2C12 suggest that EWS-FLI1 directly binds to and blocks the ability of Runx2 to induce osteoblastic differentiation [52]. Therefore, BMP pathway activation may impact on EwS biology via other transcriptional targets, most likely ID1 and ID3. Taken together, our data suggest a model in which EWS-FLI1 directly represses TGFB signaling via transcriptional down-regulation of *TGFBR2* and of down-stream targets via post-transcriptional regulation by miRNA cluster 17-92, whereas the same miRNAs inhibit *BAMBI*, potentially re-routing signaling from the TGFB to the BMP pathway.

## MATERIALS AND METHODS

### Cells and transfections

The A673/TR/shEF [53] cell line, stably carrying an sh-EWS-FLI1 construct under a doxycycline-inducible promoter was cultivated in DMEM+Glutamax (Gibco by Life Technologies, Carlsbad, CA, USA) supplemented with 10% fetal bovine serum (FBS) (10270) (Gibco), 100 U/mL penicillin, and 100 μg/mL streptomycin (Gibco) with the addition of blasticidine (2μg/ml) and zeocin (50 μg/ml) (InvivoGen, San Diego, CA, USA) at 37°C and 5% CO_2_. To efficiently knockdown EWS-FLI1, 1 μg/ml doxycycline was added to the medium for 48 h.

All other EwS cell lines used in this study have previously been described [54, 55]. Cell lines WE68, SK-N-MC, and TC252 were kindly supplied by F. Van Valen (Dept. of Pediatrics, University of Muenster, Germany), J. Biedler (Memorial Sloan-Kettering Cancer Center, New York, USA), and T. Triche (Dept. of Pathology, Children ›s Hospital, Los Angeles, USA), respectively. Cell lines were authenticated by STR testing and mycoplasm infections excluded on a regular basis (Mykoalert detection kit, LT07) (Lonza, Basel, Switzerland).

Plasmids and *mir*Vana miRNA mimics (hsa-miR-17-5p, has-miR-18a-5p and negative control mimic #1, Thermo Fisher, Waltham, MA USA) were transfected using the Lipofectamine Plus reagent (Invitrogen, Groningen, the Netherlands) according to manufacturer´s instructions.

### Plasmids

3′UTRs of *CTGF, FOSL2, SERPINE1, BAMBI, GBP3* and *RUNX3* (two parts: 287-1677 and 1677–2611) were cloned into the pmirGLO vector (Promega, Madison, USA). MiR seed sequences in the *FOSL2* and *CTGF* 3′UTR were mutated using QuikChange^®^ II Site-Directed Mutagenesis Kit (Stratagene, La Jolla, USA, 200523) according to manufacturer's instructions. Oligonucleotides containing either wildtype or mutated miRNA seed sequences for *FOSL2, SERPINE1, BAMBI, GBP3* and *RUNX3* were directly cloned into the pmirGLO vector (Promega). CMV-d2eGFP sponge plasmids for *CXCR4* and a sponge against hsa-miR-17-92 (s-α- miR-17-92) were obtained from the Jedlicka Lab [56]. pBp-Control Sponge (*CXCR4* sponge), (Addgene plasmid # 22744), and pBabe-puro-miR-9 sponge were gifts from Bob Weinberg (Addgene plasmid # 25040) [57–59]. RNA for sequencing was extracted using the RNeasy Mini Kit (Qiagen, Hilden, Germany).

### miRNA seed sequence prediction

Was performed using TargetScan (http://www.targetscan.org)

### Gene reporter assays

Cells were co-transfected with the mirGLO-based reporter constructs and pmaxEGFP (Amaxa, Cologne, Germany) using Lipofectamine Plus reagent (Invitrogen) at 20% density. Gene reporter assays were performed 48 h after transfection using the DualGlo Luciferase assay kit (Promega). EGFP positive cells and Renilla Luciferase activity were used as a measure of transfection efficiencies.

### Quantitative real-time RT-PCR

Total RNA was prepared with a Qiagen RNAeasy kit (Qiagen). cDNA was generated from 1 μg RNA (M-MLV Reverse Transcriptase, Promega) and qRT-PCR performed using Maxima^™^ SYBR Green/ROX qPCR Master Mix (Thermo Fisher). Assays were performed in triplicate using the ABI 7500 Fast Detection System (Applied Biosystems, Foster City, CA, USA). Relative expression levels were normalized to b-actin and analyzed by the 2^(−ΔΔCt)^ method [60].

### PAR-CLIP

Three PAR-CLIP experiments were performed in A673/TR/shEF. In one of these experiments EWS-FLI1 depletion was effectively achieved after 53 h doxycycline induction. PAR-CLIP was performed using an anti-AGO2 antibody kindly provided by Gunter Meister (University of Regensburg) essentially as described in [12, 61] with some small modifications. Briefly, after UV-crosslinking of A673/TR/shEF cells protein G Dynabeads (Invitrogen) were coupled to Ago2 antibody, while cells were lysed and RNA partially digested. Then AGO2 IP took place followed by RNA 3´ends dephosphorylation and linker ligation, RNA 5´ends labelling (32P-γ-ATP), and SDS-PAGE in a 4-12% NuPAGE Bis-Tris gel (Invitrogen). Protein-RNA complexes were transferred from the gel to a nitrocellulose membrane that was exposed until obtaining a clear image of the band corresponding to Ago2 molecular weight (97 kDa). This band was excised, purified and RNA was precipitated and resuspended in RNase-free H_2_O to proceed with 5´RNA linker ligation. Finally a reverse transcription step was performed, followed by PCR with Solexa flow-cell adaptors (2` 95°C, 28 cycles of 20´´ 95°C, 30´´ 58°C, 20´´ 68°C, 5´ 68°C, ∞ 4C). The PCR product was loaded in a 4% low-melt agarose gel, extracted from the gel and then eluted using the QIAquick Gel Extraction Kit (Qiagen, Hilden, Germany). DNA quantity and quality were measured using the Picogreen Assay (Invitrogen) for Nanodrop. cDNAs were then ready for sequencing.

Linkers:

3′ AUCGUAUGCCGUCUUCUGCUUGU

5′ GUUCAGAGUUCUACAGUCCGACGAUC

PCR primers:

5′ AATGATACGGCGACCACCGACAGGTTCAG

AGTTCTACAGTCCGA3′ CAAGCAGAAGACG

GCATACGA

Size-selected DNA from the PAR-CLIP experiments was sequenced on an Illumina HiSeq sequencer. Raw sequences were subsequently quality filtered and clipped (http://hannonlab.cshl.edu/fastx_toolkit, fastx_clipper -Q33 -a adaptersequence, fastq_quality_filter -Q33 -p 80 -q 25 -v) yielding at least 80 Mio high quality sequences per sample. These quality filtered sequences were aligned with bowtie to human genome version hg19 [62], (bowtie -t -v 2 -m 1 —best —strata —seed 1234 hg19) and the aligned sequences were used as input for the program PARalyzer [22] using the following parameters in the PARalyzer .ini files: BANDWIDTH=3, CONVERSION=T>C, MINIMUM_READ_COUNT_PER_GROUP=5, MINIMUM_READ_COUNT_PER_CLUSTER=5, MINIMUM_READ_COUNT_FOR_KDE=5, MINIMUM_CLUSTER_SIZE=10, MINIMUM_CONVERSION_LOCATIONS_FOR_CLUSTER=1, MINIMUM_CONVERSION_COUNT_FOR_CLUSTER=1, MINIMUM_READ_COUNT_FOR_CLUSTER_INCLUSION=5, MINIMUM_READ_LENGTH=13, #MINIMUM_READ_LENGTH=1, MAXIMUM_NUMBER_OF_NON_CONVERSION_MISMATCHES=0, EXTEND_BY_READ,MAXIMUM_SEED_MATCH_LENGTH=8.

PARalyzer identified 39929, 38646, 54341 clusters in the three experiments. For all following analyses this initial PARalyzer output was further filtered: 1) Only clusters in 3′UTRs of genes were examined. 2) Clusters were filtered to contain a miR seed sequence match not more than 3 bp up- or downstream of the location in the cluster with the highest binding signal (“ModeLocation” in the PARalyzer output). After these filtering steps 7860, 7340 and 10601 clusters were retained in the three experiments. Finally, for all seed match based analyses 6mer seed matches were excluded.

### RNA-Seq

RNA-Seq of cells transfected with sponges or treated with Noggin was performed in duplicates on an Illumina HI-Seq 2000 yielding > 16Mio 50 pb, single end reads for all samples. Short read sequencing data was quality checked using FASTQC, RNA-SeqQC (http://www.bioinformatics.babraham.ac.uk/projects), [63] and then aligned to the human genome hs37d5 (ftp://ftp.1000genomes.ebi.ac.uk/vol1/ftp/technical/reference/phase2_reference_assembly_sequence/hs37d5.fa.gz) using the STAR aligner [64] allowing for 1 mismatch and no multimappers. Further analysis was performed in R statistical environment using Bioconductor packages. Count statistics for Refseq genes were obtained by the “featureCounts” function (package “Rsubread”) and differential expression analysis was performed by edgeR and voom. All *p* values were adjusted by the Benjamini-Hochberg method [65]. For the differential gene expression analysis samples transfected with the hsa-miR-17-92 sponge or sponge against hsa-miR-9 were compared to cells transfected with the pBp-control CXCR4 sponge. For differential gene expression analysis only genes passing a cpm (counts in gene per million reads in library) cutoff of 10 in more than two samples were included.

### Data

All next generation sequencing data were submitted to GEO (GSE80494).

### Immunoblotting

Total proteins (30–50 μg) were resolved by 8.5% SDS-PAGE and processed for immunoblotting according to standard procedures. The following antibodies were used: anti-GAPDH (4300, Ambion, Thermo Fisher Scientifc, Waltham, MA, USA), anti-pSMAD1 (9516 P, Cell Signalling, Danvers, MA, USA), anti-pSMAD2 (3101 S, Cell Signalling) and anti-pSMAD3 antibody (9520 P, Cell Signalling). Linear protein quantification was performed using fluorescent dye coupled secondary antibodies (Dy LightTM800, Pierce Biotechnology, THP, Vienna, Austria) for detection by the LICOR Odyssey^®^ Infrared Imaging System (LI-COR Biosciences, Bad Homburg, Germany).

## SUPPLEMENTARY MATERIALS FIGURES AND TABLES










