# Supplementary material for: The role of miR-17-92 in the miRegulatory landscape of Ewing sarcoma
Source: Oncotarget. 2016 Dec 22;8(7):10980–93. doi: 10.18632/oncotarget.14091 (PMC5355239; doi:10.18632/oncotarget.14091)
Supplement: Supplementary file 1 [file oncotarget-08-10980-s001.pdf]

The role of miR-17-92 in the miRegulatory landscape of Ewing sarcoma

Supplementary Materials

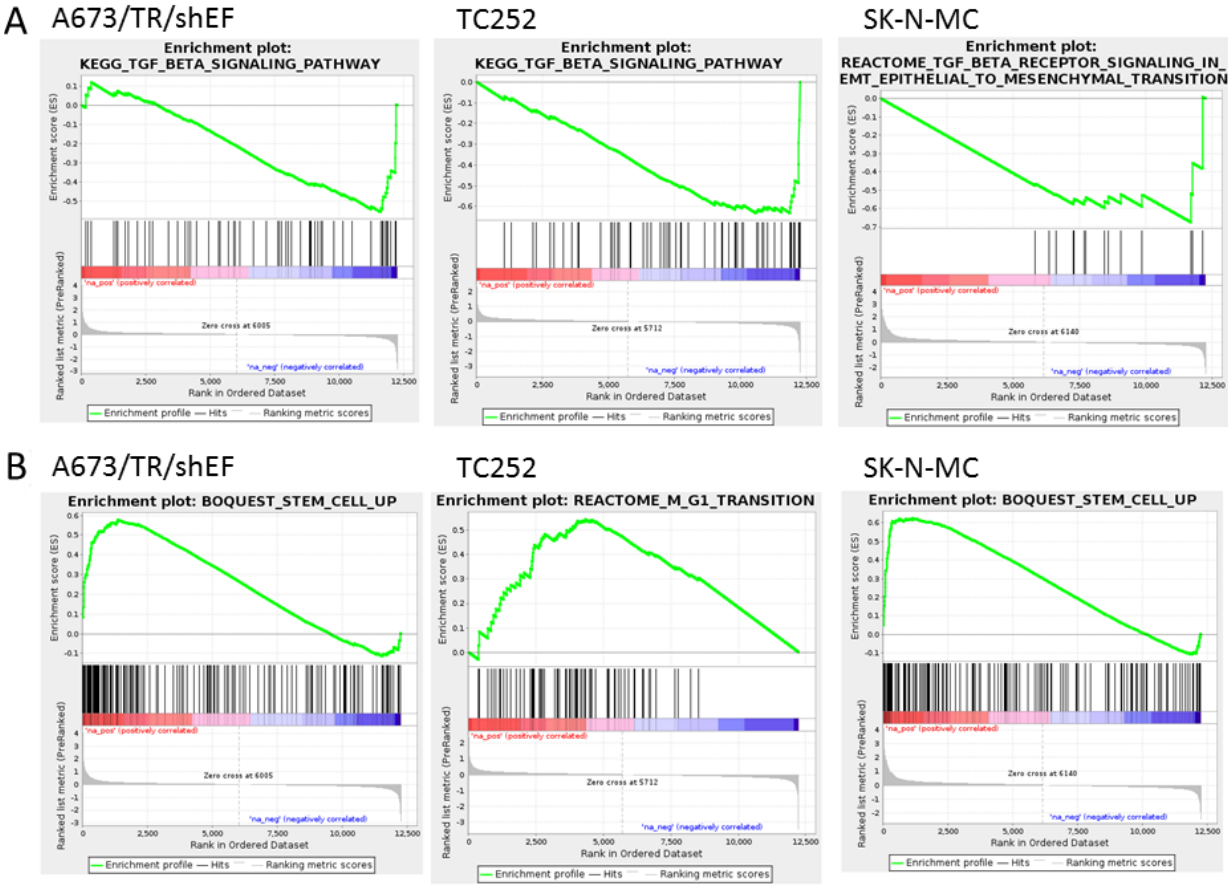

Supplementary Figure S1: GSEA enrichment plots for (**A**) Noggin suppressed genes and (**B**) genes activated by Noggin.

Supplementary Table S1: Output miRNA-Seq analysis. Differential micro-RNA analysis before and after a doxycycline induced knockdown of EWS-FLI1 in the A673 celline. Output from DESeq2. Since only one replicate was available for each condition no P values were calculated. See Supplementary_Table_S1

Supplementary Table S2: Output from the PARalyzer program for the three PAR-CLIP experiments (sheets2-4). Sheet1: Explanation of the columns in the output. See Supplementary_Table_S2

Supplementary Table S3: Overrepresentation analysis of miR seeds. (http://software.broadinstitute.org/gsea/msigdb) of 2331 genes that were identified by PARalyzer in all 3 PAR-CLIP experiments. See Supplementary_Table_S3

Supplementary Table S4: Results from Sponge and PAR-CLIP analysis. Sheet1: Differential gene expression matrix with logFC and P-values for the 3 different Sponge experiments (Sponge 17-92, Sponge-9) from RNA-Seq. Differential gene expression is measured against the CXCR4 control. Sheet2: 87 genes that were up-regulated by Sponge 17~92 AND contained a PAR-CLIP cluster containing a 17~92 seed match. Sheet3/Sheet4: Annotation of the 87 genes by MSigDb and DAVID (http://software.broadinstitute.org/gsea/msigdb, https://david.ncifcrf.gov). See Supplementary_Table_S4

Supplementary Table S5: Genes from combined PAR-CLIP and sponge analysis for miR-17-92 annotate preferentially to TGFB/BMP pathway

syms		TGF	BMP
CTGF	regulated by TGFB	(1)	
FOSL2	regulated by TGFB	(2)	
MXD1	regulated by TGFB	(3)	
TRAM2	BMP-RUNX2-TRAM2		(4)
BAMBI	BMP pseudoreceptor, binds and inhibits BMPtypeII receptors	(5)	
RUNX3	integral component of TGF/BMP signaling; SMAD co-factor	(6)	
ADAM19	regulated by TGFB	(7), (8), (9)	
KLF10	TGFB inducible	(10)	
DUSP5	inactivates ERK2, which negativbely regulates TGFB signaling	(11), (12)	
SOX4	transcriptional target of TGFB and BMP2 signaling	(13), (14)	(15)
CDKN1A	induced by TGFB and BMPs2&4	(16), (17)	(18)
EEA1	Clathrin-dependent internalization into the EEA1-positive endosome, where the Smad2 anchor SARA is enriched, promotes TGFB signalling	(19), (20)	
TSC1	TSC1 interacts with the TGFB receptor complex and Smad2/3 and is required for their association with one another.	(21)	
MAML1	Notch activation inhibits TGFB/Smad1 and TGFB/Smad2 signaling pathways by decreasing the expression of Smad1 and Smad2 and their target genes	(22)	
SOX11	Binds and activates SMAD3, TGFBR1, 12 others in TGFB pathway	(23)	
F3	BMP7 induces F3 via NFKB		(24)
AZIN1	negative regulator of TGFB/SMAD signaling	(25)	
FRS2	BMP blocker noggin inhibits the canonical FGF-to-ERK pathway upstream of FRS2 activation and also prevents FGF from stimulating FRS2- and ERK- independent gene expression, indicating that BMP signaling is required at the level of FGF receptors		(26)
ELK3	TGFB1 significantly increased endogenous Elk-3 mRNA levels that had been down-regulated by LPS in macrophages	(27)	
MAPK8	induced by TGFB	(28), (29)	

1. Parada C, Li J, Iwata J, Suzuki, A, Chai Y. CTGF mediates Smad-dependent transforming growth factor beta signaling to regulate mesenchymal cell proliferation during palate development. Mol Cell Biol. 2013; 33:3482–3493.
2. Wang J, Sun D, Wang Y, Ren F, Pang S, Wang D, Xu S. FOSL2 positively regulates TGF-beta1 signalling in non-small cell lung cancer. PLoS ONE. 2014; 9:e112150.
3. Siegel PM, Shu, W, Massague J. Mad upregulation and Id2 repression accompany transforming growth factor (TGF)-beta-mediated epithelial cell growth suppression. J Biol Chem. 2003; 278:35444–5450.
4. Pregizer S, Barski A, Gersbach CA, Garcia AJ, Frenkel, B. Identification of novel Runx2 targets in osteoblasts: cell type-specific BMP-dependent regulation of Tram2. J Cell Biochem. 2007; 102:1458–1471.
5. Onichtchouk D, Chen YG, Dosch R, Gawantka V, Delius H, Massague, J, Niehrs C. Silencing of TGF-beta signalling by the pseudoreceptor BAMBI. Nature. 1999; 401:480–485.
6. Lee CW, Ito, K, Ito Y. Role of RUNX3 in bone morphogenetic protein signaling in colorectal cancer. Cancer Res. 2010; 70:4243–4252.

7. Ramdas V, McBride M, Denby, L, Baker AH. Canonical transforming growth factor-beta signaling regulates disintegrin metalloprotease expression in experimental renal fibrosis via miR-29. Am J Pathol. 2013; 183:1885–1896.
8. Chan MW, Huang YW, Hartman-Frey C, Kuo CT, Deatherage D, Qin H, Cheng AS, Yan PS, Davuluri RV, Huang TH. et al. Aberrant transforming growth factor beta1 signaling and SMAD4 nuclear translocation confer epigenetic repression of ADAM19 in ovarian cancer. Neoplasia. 2008; 10:908–919.
9. Keating DT, Sadlier DM, Patricelli A, Smith SM, Walls D, Egan JJ, Doran PP. Microarray identifies ADAM family members as key responders to TGF-beta1 in alveolar epithelial cells. Respir Res. 2006; 7:114.
10. Subramaniam M, Harris SA, Oursler MJ, Rasmussen K, Riggs BL, Spelsberg TC. Identification of a novel TGF-beta-regulated gene encoding a putative zinc finger protein in human osteoblasts. Nucleic Acid Res. 1995; 23:4907–4912.
11. Mandl M, Slack DN, Keyse SM. Specific inactivation and nuclear anchoring of extracellular signal-regulated kinase 2 by the inducible dual-specificity protein phosphatase DUSP5. Mol Cell Biol. 2005; 25:1830–1845.
12. Kosla J, Dvorakova M, Dvorak, M, Cermak V. Effective myofibroblast dedifferentiation by concomitant inhibition of TGF-beta signaling and perturbation of MAPK signaling. Eur J Cell Biol. 2013; 92:363–373.
13. Vervoort SJ, Lourenco AR, van Boxtel, R, Coffer PJ. SOX4 mediates TGF-beta-induced expression of mesenchymal markers during mammary cell epithelial to mesenchymal transition. PLoS ONE. 2013; 8:e53238.
14. Kuwahara M, Yamashita M, Shinoda K, Tofukuji S, Onodera A, Shinnakasu R, Motohashi S, Hosokawa H, Tumes D, Iwamura C, Lefebvre V, Nakayama T. The transcription factor Sox4 is a downstream target of signaling by the cytokine TGF-beta and suppresses T(H)2 differentiation. Nat Immunol. 2012; 13:778–786.
15. Locklin RM, Riggs BL, Hicok KC, Horton HF, Byrne MC, Khosla, S. Assessment of gene regulation by bone morphogenetic protein 2 in human marrow stromal cells using gene array technology. J Bone Miner Res. 2001; 16:2192–2204.
16. Datto MB, Yu Y, Wang XF. Functional analysis of the transforming growth factor beta responsive elements in the WAF1/Cip1/p21 promoter. J Biol Chem. 1995; 270:28623–28628.
17. Pardali K, Kurisaki A, Moren A, ten Dijke P, Kardassis, D, Moustakas A. Role of Smad proteins and transcription factor Sp1 in p21(Waf1/Cip1) regulation by transforming growth factor-beta. J Biol Chem. 2000; 275:29244–29256.
18. Jernvall J, Aberg T, Kettunen P, Keranen, S, Thesleff I. The life history of an embryonic signaling center: BMP-4 induces p21 and is associated with apoptosis in the mouse tooth enamel knot. Development. 1998; 125:161–169.
19. Di Guglielmo GM, Le Roy C, Goodfellow AF, Wrana JL. Distinct endocytic pathways regulate TGF-beta receptor signalling and turnover. Nat Cell Biol. 2003; 5:410–421.
20. Hayes S, Chawla, A, Corvera S. TGF beta receptor internalization into EEA1-enriched early endosomes: role in signaling to Smad2. J Cell Biol. 2002; 158:1239–1249.
21. Thien A, Prentzell MT, Holzwarth B, Klasener K, Kuper I, Boehlke C, Sonntag AG, Ruf S, Maerz L, Nitschke R, Grellscheid SN, Reth M, Walz G, et al. TSC1 activates TGF-beta-Smad2/3 signaling in growth arrest and epithelial-to-mesenchymal transition. Dev Cell. 2015; 32:617–630.
22. Fu Y, Chang A, Chang L, Niessen K, Eapen S, Setiadi, A, Karsan A. Differential regulation of transforming growth factor beta signaling pathways by Notch in human endothelial cells. J Biol Chem. 2009; 284:19452–19462.
23. Kuo PY, Leshchenko VV, Fazzari MJ, Perumal D, Gellen T, He T, Iqbal J, Baumgartner-Wennerholm S, Nygren L, Zhang F, Zhang W, Suh KS, Goy A, et al. High-resolution chromatin immunoprecipitation (ChIP) sequencing reveals novel binding targets and prognostic role for SOX11 in mantle cell lymphoma. Oncogene. 2015; 34:1231–1240.
24. Sovershaev TA, Egorina EM, Unruh D, Bogdanov VY, Hansen JB, Sovershaev MA. BMP-7 induces TF expression in human monocytes by increasing F3 transcriptional activity. Thromb Res. 2015; 135:398–403.
25. Li R, Chung AC, Dong Y, Yang W, Zhong, X, Lan HY. The microRNA miR-433 promotes renal fibrosis by amplifying the TGF-beta/Smad3-Azin1 pathway. Kidney Int. 2013; 84:1129–1144.
26. Boswell BA, Musil LS. Synergistic Interaction Between the Fibroblast Growth Factor and Bone Morphogenetic Protein Signaling Pathways in Lens Cells. Mol Biol Cell. 2015.
27. Chen YH, Layne MD, Chung SW, Ejima K, Baron RM, Yet SF, Perrella MA. Elk-3 is a transcriptional repressor of nitric-oxide synthase 2. J Biol Chem. 2003; 278:39572–39577.
28. Sajid M, Lele, M, Stouffer GA. Autocrine thrombospondin partially mediates TGF-beta1- induced proliferation of vascular smooth muscle cells. Am J Physiol Heart Circ Physiol. 2000; 279:H2159–2165.
29. Chang, Y, Wu XY. JNK1/2 siRNA inhibits transforming-growth factor-beta1-induced connective tissue growth factor expression and fibrotic function in THSFs. Mol Cell Biochem. 2010; 335:83–8.

Supplementary Table S6: Previously described miR-17-92 targets in combined PAR-CLIP and sponge analysis

syms	known miR regulation	Ref
CTGF	mir-19	(1)
MXD1	mir-19/ab	(2)
BAMBI	mir-20a	(3)
ABL2	mir-20a	(4)
KDM2A	miR-19b	(5)
CDKN1A	mir-20a	(6)
MAPK8	miR-92	(7)

1. van Almen GC, Verhesen W, van Leeuwen RE, van de Vrie M, Eurlings C, Schellings MW, Swinnen M, Cleutjens JP, van Zandvoort MA, Heymans S, Schroen B. MicroRNA-18 and microRNA-19 regulate CTGF, TSP-1 expression in age-related heart failure. Aging cell. 2011; 10:769–779.
2. Wu Q, Yang Z, An Y, Hu H, Yin J, Zhang P, Nie Y, Wu K, Shi Y, Fan D. MiR-19a/b modulate the metastasis of gastric cancer cells by targeting the tumour suppressor MXD1. Cell Death Dis. 2014; 5:e1144.
3. Zhang JF, Fu WM, He ML, Xie WD, Lv Q, Wan G, Li G, Wang H, Lu G, Hu X, Jiang S, Li JN, Lin MC, et al. MiRNA-20a promotes osteogenic differentiation of human mesenchymal stem cells by co-regulating BMP signaling. RNA Biol. 2011; 8:829–838.
4. Qiang XF, Zhang ZW, Liu Q, Sun N, Pan LL, Shen J, Li T, Yun C, Li H, Shi LH. miR-20a promotes prostate cancer invasion and migration through targeting ABL2. J Cell Biochem. 2014; 115:1269–1276.
5. Gantier MP, Stunden HJ, McCoy CE, Behlke MA, Wang D, Kaparakis-Liaskos M, Sarvestani ST, Yang YH, Xu D, Corr SC, Morand EF, Williams BR. A miR-19 regulon that controls NF-kappaB signaling. Nucleic Acids Res. 2012; 40:8048–8058.
6. Sokolova V, Fiorino A, Zoni E, Crippa E, Reid JF, Gariboldi, M, Pierotti MA. The Effects of miR-20a on p21: Two Mechanisms Blocking Growth Arrest in TGF-beta Responsive Colon Carcinoma. J Cell Physiol. 2015.
7. Zhang L, Zhou M, Wang Y, Huang W, Qin G, Weintraub NL, Tang, Y. miR-92a inhibits vascular smooth muscle cell apoptosis: role of the MKK4-JNK pathway. Apoptosis. 2014; 19:975–983.

Supplementary Table S7: Top 20 consistently down-regulated genes upon 16 hours Noggin treatment with logFC and P-values for the 3 different EwS cell lines SK-N-MC, TC252 and A673/TR/shEF from RNA-Seq (experiments were performed in duplicates). Differential gene expression is measured against the untreated control.

Gene Symbol	Entrez Gene	logFC_sk_ nog_vs_ctrl	logFC_tc_ nog_vs_ctrl	logFC_A673_ nog_vs_ctrl	adjP_sk_ nog_vs_ctrl	adjP_tc_ nog_vs_ctrl	adjP_A673_ nog_vs_ctrl
OCA2	4948	−0.51	−2.18	−2.08	0.2462	0.8923	0.9747
NOG	9241	−0.51	−1.02	−1.37	0.4334	9.00E-04	0.6947
ID1	3397	−0.85	−1.2	−1.19	0.126	1.00E-04	0
**ID3**	**3399**	**−1.25**	**−1.19**	**−0.98**	**0.0025**	**9.00E-04**	**0.0038**
METTL7B	196410	−0.68	−0.92	−0.97	0.0791	0.9535	0.0045
GPER1	2852	−0.4	−1.31	−0.82	0.8983	0.9535	0.4127
SLC43A2	124935	−0.18	−1.53	−0.42	0.7612	0.9701	0.9998
TM6SF1	53346	−0.12	−1.31	−0.36	0.6846	0.9535	0.9998
KCNC4	3749	−0.65	−1.22	−0.33	0.0661	0.9535	0.9998
ACE	1636	−0.34	−1.2	−0.27	0.222	0.9743	0.9998
TCEB3−AS1	100506963	−0.22	−0.95	−0.26	0.7698	0.9535	0.9998
NTSR1	4923	−0.67	−2.01	−0.18	0.2864	0.9535	0.7888
ETV4	2118	−0.64	−2.63	−0.11	0.8798	0.9535	0.9998
TFEC	22797	−0.24	−1.06	−0.04	0.9047	0.9535	0.9998
PADI1	29943	−0.2	−0.7	0.03	0.8955	0.9757	0.9998
PCDHA10	56139	−1.27	−0.85	0.08	0.3268	0.9835	0.9998
INPP4B	8821	−0.45	−0.77	0.12	0.7997	0.9757	0.9998
SLC7A3	84889	−0.24	−0.72	0.13	0.9736	0.9535	0.9998
STK32C	282974	−0.26	−1.17	0.16	0.7701	0.9535	0.9998
